# Physical rehabilitation interventions for adult patients with critical illness across the continuum of recovery: an overview of systematic reviews protocol

**DOI:** 10.1186/s13643-015-0119-y

**Published:** 2015-09-29

**Authors:** Bronwen Connolly, Brenda O’Neill, Lisa Salisbury, Kathryn McDowell, Bronagh Blackwood, on behalf of the Enhanced Recovery After Critical Illness Programme Group

**Affiliations:** Lane Fox Clinical Respiratory Physiology Research Unit, Guy’s and St. Thomas’ NHS Foundation Trust, London, UK; Centre of Human and Aerospace Physiological Sciences, King’s College London, London, UK; Guy’s & St Thomas’ NHS Foundation Trust and King’s College London, National Institute of Health Research Biomedical Research Centre, London, UK; School of Health Sciences, Institute of Nursing and Health Research, Ulster University, Ulster, UK; School of Health in Social Science, The University of Edinburgh, Edinburgh, UK; School of Medicine, Dentistry and Biomedical Sciences, Queen’s University Belfast, Belfast, UK

**Keywords:** Critical illness, Physical rehabilitation, Recovery, Systematic review, Quality

## Abstract

**Background:**

Patients admitted to the intensive care unit with critical illness often experience significant physical impairments, which typically persist for many years following resolution of the original illness. Physical rehabilitation interventions that enhance restoration of physical function have been evaluated across the continuum of recovery following critical illness including within the intensive care unit, following discharge to the ward and beyond hospital discharge. Multiple systematic reviews have been published appraising the expanding evidence investigating these physical rehabilitation interventions, although there appears to be variability in review methodology and quality. We aim to conduct an overview of existing systematic reviews of physical rehabilitation interventions for adult intensive care patients across the continuum of recovery.

**Methods/design:**

This protocol has been developed according to the Preferred Reporting Items for Systematic Reviews and Meta-Analyses Protocol (PRISMA-P) guidelines. We will search the Cochrane Systematic Review Database, Database of Abstracts of Reviews of Effectiveness, Cochrane Central Register of Controlled Trials, MEDLINE, Excerpta Medica Database and Cumulative Index to Nursing and Allied Health Literature databases. We will include systematic reviews of randomised controlled trials of adult patients, admitted to the intensive care unit and who have received physical rehabilitation interventions at any time point during their recovery. Data extraction will include systematic review aims and rationale, study types, populations, interventions, comparators, outcomes and quality appraisal method. Primary outcomes of interest will focus on findings reflecting recovery of physical function. Quality of reporting and methodological quality will be appraised using the PRISMA checklist and the Assessment of Multiple Systematic Reviews tool.

**Discussion:**

We anticipate the findings from this novel overview of systematic reviews will contribute to the synthesis and interpretation of existing evidence regarding physical rehabilitation interventions and physical recovery in post-critical illness patients across the continuum of recovery.

**Systematic review registration:**

PROSPERO CRD42015001068.

## Background

Admission to the intensive care unit (ICU) with critical illness is typically associated with significant morbidity for survivors, including profound impairment in physical strength and functional performance. These symptoms contribute to the ‘post-intensive care syndrome’ evident in survivors of critical illness [1] and have been shown to persist up to 5 years following resolution of the original illness [2–7]. Peripheral skeletal muscle wasting and dysfunction that occurs early and rapidly during critical illness [8] contributes to the development of intensive care unit-acquired weakness (ICU-AW) and these physical sequelae.

Physical rehabilitation interventions are advocated to address physical and functional deficits associated with ICU-AW, and delivery is advocated across the continuum of the patient pathway commencing in the ICU, following transfer to the ward and beyond hospital discharge into the community [9]. Typically, early mobilisation of patients in the ICU represents a hierarchical progression of increasingly functional activities such as active-assisted bed exercises, sitting on the edge of the bed, standing, marching-on-the-spot and walking [10], with use of adjunctive technologies including electrical muscle stimulation [11], interactive video games [12] and passive cycle ergometry [13]. On ICU discharge to the ward, the emphasis of physical rehabilitation for post-critical illness patients is directed towards the necessary level of functional mobility required to expedite hospital discharge, which can be supplemented by input from generic rehabilitation assistants [14, 15]. Following hospital discharge, physical rehabilitation has been evident in the delivery of either home- or hospital-based exercise rehabilitation programmes including combined strength, cardiovascular and functional components [16–19].

As the profile of physical rehabilitation for critical illness patients has increased and the volume of published data exploring the effectiveness of interventions has grown, multiple systematic reviews have been reported aiming to synthesise available evidence and draw conclusions on the most beneficial therapeutic options. A number of these have focused on interventions delivered within the ICU, e.g. Kayambu et al. [20], Calvo-Ayala et al. [21], Li et al. [22] and Stiller [23], albeit the stages of recovery following ICU discharge are also being evaluated [24, 25]. These systematic reviews are valuable for summarising findings from clinical trials at discrete time points along the recovery trajectory, involving particular types of physical rehabilitation strategies, in defined ICU populations and evaluating impact on select outcomes. However, this specificity of focus fails to provide a broader overview of the existing evidence base for physical rehabilitation during recovery from critical illness spanning the acute to chronic phases, to elucidate patient cohorts that may respond to interventions at different time points, and temporal variation in intervention effectiveness. In addition, variability in individual review methodology and quality appears evident which can influence robustness and clinical applicability of findings.

### Objectives of this overview

We will carry out an overview of existing Systematic review (SR) evaluating physical rehabilitation interventions for adult patients with critical illness patients across the continuum of recovery. Variability in the review question and population, intervention, comparator and outcome components across the large body of currently available reviews is a limiting factor to the overall synthesis of their findings and translation into clinical practice. An overview of SR offers a logical and appropriate step allowing comparison and contrast of individual reviews to be made and providing a precis of evidence at these different levels [26].

Undertaking an overview of existing reviews is a novel approach within the field of physical rehabilitation following critical illness. Adopting this strategy, our aim is to summarise and appraise the best available evidence for the use of physical rehabilitation interventions across the continuum of recovery following critical illness, to address the following questions:

1. At which stage of the post-critical illness recovery continuum (within the ICU, following ICU discharge on the ward and post-hospital discharge) do physical rehabilitation interventions have the most effect?
2. Do particular patient populations gain more benefit from post-critical illness physical rehabilitation interventions than others?
3. Which type of physical rehabilitation interventions produce benefits (short- and long-term) for post-critical illness patients?
4. What adverse events or harmful effects are experienced from receipt of any physical rehabilitation interventions?

## Methods/design

### Protocol and registration

Our protocol has been written using the Preferred Reporting Items for Systematic Reviews and Meta-Analyses Protocol (PRISMA-P) [27] and is registered on PROSPERO (CRD42015001068).

### Data sources and search strategy

We will search the Cochrane Systematic Review Database, Database of Abstracts of Reviews of Effectiveness, Cochrane Central Register of Controlled Trials (CENTRAL), Ovid SP MEDLINE, Ovid SP Excerpta Medica Database (EMBASE) and Cumulative Index to Nursing and Allied Health Literature (CINAHL) via EBSCO host. All authors contributed to devising the search strategies for each database using a combination of subject headings and free-text keywords to describe critical illness, physical rehabilitation interventions and the different stages of the recovery continuum. Table 1 presents the search strategy for use in Ovid SP MEDLINE. No date or language restrictions will be applied to the initial searches.

**Table 1:** Example search strategy for Ovid SP MEDLINE

1. ((intensive or critical) adj1 care unit).mp. [mp=title, abstract, original title, name of substance word, subject heading word, keyword heading word, protocol supplementary concept word, rare disease supplementary concept word, unique identifier]	
2. ((intensive or critical) adj1 care).mp. [mp=title, abstract, original title, name of substance word, subject heading word, keyword heading word, protocol supplementary concept word, rare disease supplementary concept word, unique identifier]	
3. exp Intensive Care Units/ or exp Intensive Care/ or ICU.mp. or exp Critical Care/	
4. critical illness.mp. or exp Critical Illness/	
5. mechanical ventilation.mp. or exp Respiration, Artificial/	
6. 1 or 2 or 3 or 4 or 5	
7. exp Exercise Therapy/ or exercise.mp. or exp Exercise/	
8. ((exercise or physical) adj2 rehabilitation).mp. [mp=title, abstract, original title, name of substance word, subject heading word, keyword heading word, protocol supplementary concept word, rare disease supplementary concept word, unique identifier]	
9. physiotherapy.mp.	
10. physical therapy.mp.	
11. exp Early Ambulation/ or early mobilisation.mp.	
12. early mobilization.mp.	
13. physical fitness.mp. or exp Physical Fitness/	
14. muscle strength.mp. or exp Muscle Strength/	
15. cycling.mp.	
16. cycle ergometry.mp.	
17. exp Electric Stimulation Therapy/ or electrical muscle stimulation.mp.	
18. neuromuscular stimulation.mp.	
19. NMES.mp.	
20. 7 or 8 or 9 or 10 or 11 or 12 or 13 or 14 or 15 or 16 or 17 or 18 or 19	
21. randomized controlled trial.pt.	
22. controlled clinical trial.pt.	
23. randomized.ab.	
24. placebo.ab.	
25. randomly.ab.	
26. trial.ab.	
27. groups.ab.	
28. 21 or 22 or 23 or 24 or 25 or 26 or 27	
29. exp animals/ not humans.sh.	
30. 28 not 29	
31. Meta-Analysis as Topic/	
32. meta analy$.tw.	
33. metaanaly$.tw.	
34. Meta-Analysis/	
35. (systematic adj (review$1 or overview$1)).tw.	
36. exp Review Literature as Topic/	
37. or/31-36	
38. cochrane.ab.	
39. embase.ab.	
40. (cinahl or cinhal).ab.	
41. science citation index.ab.	
42. or/38-41	
43. reference list$.ab.	
44. bibliograph$.ab.	
45. hand-search$.ab.	
46. relevant journals.ab.	
47. manual search$.ab.	
48. or/43-47	
49. selection criteria.ab.	
50. data extraction.ab.	
51. 49 or 50	
52. Review/	
53. 51 and 52	
54. Comment/	
55. Letter/	
56. Editorial/	
57. animal/	
58. human/	
59. 57 not (57 and 58)	
60. or/54-56,59	
61. 37 or 42 or 48 or 53	
62. 61 not 60	
63. 6 and 20 and 30	
64. 6 and 20 and 62	

### Selection of reviews

Following initial removal of duplicate and non-relevant material, two review authors will independently screen search results (based on abstract and title) against inclusion criteria (see below) for full-text review (BC and LS initial searches; BC and BO’N for updated searches). Full-text papers will then be further screened for eligibility. A bespoke assessment of study eligibility form will be used for documentation. In the absence of consensus, arbitration by a third author (KM) will be sought. References will be managed in Endnote v7.0 (Thomson Reuters).

### Types of reviews

We will include systematic reviews of randomised controlled trials (RCTs) investigating the effect of any physical rehabilitation interventions following critical illness at any stage of the recovery continuum. If a review includes quasi-RCTs in addition to RCTs, we will also include these. Where non-RCTS are included in the review, the review will be included only if the RCTs are reported separately.

### Types of participants

Participants will be adult patients who have been admitted to the ICU with critical illness, irrespective of causal diagnosis. We will exclude systematic reviews that only include studies of short-stay post-operative management (less than 48-h length of stay in the ICU). This is to focus the review content on patients considered most likely to develop intensive care unit-acquired weakness and go on to experience the physical consequences of critical illness and require physical rehabilitation interventions. Data extraction will include details of the populations included in each eligible systematic review, and these can be discussed and interpreted accordingly.

### Types of intervention

We will include all types of physical rehabilitation interventions including exercise-based treatments and adjunctive strategies such as electrical muscle stimulation or cycling. These interventions can either be compared to control interventions (standard or usual care) or an alternative physical rehabilitation. We will exclude reviews including studies of composite interventions such as combined physical and cognitive rehabilitation and reviews including studies where the comparator was the same intervention but delivered at a different level of intensity.

### Types of outcomes

#### Primary outcomes

Primary outcomes of interest are findings reflecting the recovery of any aspect of physical function, long-term measures of physical function or its surrogates. Where applicable, these outcomes will be summarised according to type of physical rehabilitation intervention, e.g. exercise-based rehabilitation programmes or electrical stimulation, and time point of assessment and duration of follow-up (such as 1, 3 or 12 months post-ICU discharge).

#### Secondary outcomes

Secondary outcomes will include the following: structure, content and format of physical rehabilitation interventions according to recovery continuum stage; details on specific patient populations examined in each included review; reported rates of adverse events or harmful effects associated with interventions; and effect on other domains of outcome such as health-related quality of life where examined and reported.

### Data extraction and analysis

Data extraction and analysis will be conducted in line with guidance from the Cochrane Handbook of Systematic Reviews of Interventions. Full texts of included reviews will be retrieved. Two review authors (LS and BO’N) will independently extract descriptive and outcome data from each included review. A third review author (KM) will arbitrate in the event that discrepancies cannot be resolved by consensus. A bespoke data extraction form will be designed, piloted and used to record review features including the aims and rationale, types of studies included, population(s), intervention(s), comparator(s), outcomes (including beneficial and harmful effects and reported adverse events) and date of last search and methods of assessing quality of studies. In the event that we include more than one review containing the same studies, we will examine the review question and comparisons explored, the date of the last search and key aspects of methodological quality (e.g. types of studies included and risk of bias assessment) and will list the individual studies included in each of the review. In this way, we will enable identification of trials included in one review but not another. Using these data, we will determine which of the reviews should contribute data to the results based on the review with the more current search strategy and more recent trials.

### Assessment of methodological quality of included reviews

Two review authors (BC and BB) will independently assess the quality of reporting and the methodological quality of included reviews using the Preferred Reporting Items for Systematic Reviews and Meta-Analyses (PRISMA) [28] checklist and the Assessment of Multiple Systematic Reviews (AMSTAR) tool [29], respectively. We will report the proportion of PRISMA quality (number of items reported/27 checklist items*100 %). We will deem systematic reviews achieving an AMSTAR score of 8 to 11 of high methodological quality, 4 to 7 of medium quality and 0 to 3 as low quality [30].

We will use the Grading of Recommendations Assessment, Development and Evaluation (GRADE) approach [31] to assess the overall quality of evidence within, and across, the systematic reviews for each outcome. Disagreement will be resolved through consensus, and where this is not achieved, arbitration by a third review author (KM) will be sought.

### Dealing with missing data

Reasons for missing data will be recorded as reported by the original reviews. If the original reviews included this detail, we will report the number of studies that performed intention-to-treat or per protocol analyses.

### Data synthesis and reporting

Data will be presented as a narrative synthesis, with textual commentary supplemented with use of summary tables and figures to enhance clarity of reporting [26]. We will document primary and secondary outcomes of each intervention comparison in an included review, as well as the number of studies and number of participants included in the comparison, and (when reported in reviews) the mean difference (or standardised mean difference), 95 % confidence intervals and *I*^2^ statistic for heterogeneity [32]. We will synthesise key information pertaining to the quality of evidence, and documented eligibility criteria, study characteristics and the primary outcome of each review. As previously described, we will also use the GRADE approach to determine overall quality of evidence and methodological checklist items for standardised evaluation of reporting and quality appraisal [28, 29]. Flow diagrams will be used to summarise the study selection process. Finally, reasons for excluding reviews will be reported.

### Sensitivity analysis

If applicable, we will conduct sensitivity analysis in relation to studies of differing methodological quality.

### Sub-group analysis

Depending on sufficiency of reviews, we plan to analyse the main functional outcomes according to patient population (e.g. age and causal diagnosis), intervention (i.e. type of physical intervention) and setting (i.e. within ICU, within the ward, post-hospital discharge).

## Discussion

### Expected significance of the study

The findings of this overview of systematic reviews of physical rehabilitation for adult patients with critical illness across the continuum of recovery will potentially have implications for clinical practice, research and future guideline development and update. It is intended that our results provide greater clarity and synthesis of available evidence on the most effective physical rehabilitation interventions to deliver according to the stage of recovery following critical illness. This is likely to have impact on existing and planned resource allocation in the clinical environment, inform the direction of future research including randomised controlled trials of intervention effectiveness and underpin guideline recommendations. Conclusions from this overview will highlight which, if any, physical rehabilitation interventions demonstrate clear benefit and those for which there is no clear evidence at which, if any, stage in the continuum of recovery. If sufficient data are available, our findings may also add clarity to the ‘dose’ of physical rehabilitation intervention, i.e. type, timing, intensity, duration and also circumstances under which any adverse effects of harm was caused as a result of the intervention.

### Potential limitations of overview design

We plan to follow the approach outlined by the Cochrane Collaboration for undertaking an overview [33]. As such, including individual studies that have not previously been included in a systematic review is beyond the scope of an overview although this could be considered a limitation if any new evidence exists. We will note when included systematic reviews are out of date and identify any relevant new studies that have been published subsequent to the date of the last reported systematic review search although we will not formally undertake a new systematic review within our overview framework [33]. Our discussion will focus on the current state of the systematic review evidence for physical rehabilitation interventions for adults with critical illness, albeit we will comment on the volume of outstanding evidence identified as awaiting inclusion in systematic review.

### Conclusion

It is intended that this overview will provide insight regarding considerations for the design and conduct of future interventional trials of physical rehabilitation intervention in the post-critical illness population, acknowledged to be complex studies [34].

## Abbreviations

AMSTAR: Assessment of Multiple Systematic Reviews
GRADE: Grading of Recommendations Assessment, Development and Evaluation
ICU: Intensive care unit
ICU-AW: Intensive care unit-acquired weakness
PRISMA: Preferred Reporting Items for Systematic Reviews and Meta-Analyses
RCT: Randomised controlled trial
